# Hélio Pereira de Magalhães

**DOI:** 10.5935/1678-9741.20160052

**Published:** 2016

**Authors:** Maurício Galantier

**Affiliations:** 1 Graduated from the Universidade Federal do Paraná of Medical School in 1963

He became interested in surgery during his undergraduate studies, particularly in experimental surgery (general and cardiovascular) and cardiopulmonary bypass (under the supervision of Dante Romanó).

He did his residency in Cardiovascular Surgery at the Instituto de Cardiologia do Estado de São Paulo (ICE), currently, the Institute Dante Pazzanese de Cardiologia, in 1964 and 1965, remaining at that institution until 1969.

A competent, careful, and highly skilled surgeon, he stood out for his ideas and experiments aimed at facilitating and improving surgical procedure outcomes.

He was in charge of the Experimental Surgery service of ICE, bringing his experience from Curitiba in showing the same consideration for the experimental animal as he would for a patient, doing postoperative care and following them up to "discharge".

He was the main contributor to the development of the ICE model of a vertical bubble oxygenator, which incorporated an oxygenating column, a defoamer, a reservoir, and a heat exchanger in a single device. That model, with its variations and improvements, was the most used from 1964 to the emergence of the hollow-fiber oxygenators.

Likewise, he was responsible for developing the Starr-Edwards mechanical prostheses at the ICE, also one of the most used in our midst for many years. After leaving the ICE, he created Indústria Macchi, which brought numerous contributions not only to cardiovascular surgery (cardiopulmonary bypass machines, oxygenators, cardiac prostheses, tubes and accessories kit, support for bioprosthesis, among others), but also to neuro (hydrocephalus valves) and bariatric surgery (gastric balloon). Macchi was later bought by Edwards. Then, he founded another company, the HP Bio, which remains active under the control of his children. At HP Bio, he developed the Biplus bileaflet carbon cardiac valve prosthesis.

He defended his doctoral thesis at the Escola Paulista de Medicina (currently, Universidade Federal de São Paulo - UNIFESP) in 1977, entitled "Bubble oxygenator with heat exchanger for cardiopulmonary bypass: clinical applications, assessment of performance and capacity".

Besides several activities in cardiovascular surgery, he had a brilliant teaching career at the Organização Santamarense de Educação e Cultura (UNISA) Medical School in the Morphology, Thoracic and Cardiovascular Surgery, and Surgical Technique chairs, where he organized one of the best courses in this subject among the medical schools. Thanks to his dedication and teaching standards, he received several honors and awards. He was also a patron of the 7^th^ graduating class at UNISA, in 1983. During his teaching career, he published an excellent book on Surgical Technique and Experimental Surgery, used as reference in many colleges, as well as the *Princípios de Radiologia do Coração e dos Grandes Vasos da Base* (Principles of Radiology of the Heart and Great Vessels) book.

It is with great sadness that we announce his passing on May 27, 2016. This memorial is an important reminder to his many pupils, as an example of dedication, as well as to the younger surgeons, who surely apply the legacy of this distinguished surgeon to their surgeries.

